# Electroencephalography Can Distinguish between Pain and Anaesthetic Intervention in Conscious Lambs Undergoing Castration

**DOI:** 10.3390/ani10030428

**Published:** 2020-03-04

**Authors:** Charissa Harris, Peter John White, Virginia L. Mohler, Sabrina Lomax

**Affiliations:** 1 School of Life and Environmental Sciences, Faculty of Science, The University of Sydney, Sydney 2006, Australia; char2922@uni.sydney.edu.au; 2 Sydney School of Veterinary Science, Faculty of Science, The University of Sydney, Sydney 2006, Australia; p.white@sydney.edu.au (P.J.W.); jennie.mohler@sydney.edu.au (V.L.M.)

**Keywords:** Electroencephalography, castration, anaesthesia, sheep, pain

## Abstract

**Simple Summary:**

Australian sheep undergo painful procedures such as castration as part of routine husbandry practices, usually without any pain relief. Pain can be difficult to measure in prey animals like sheep due to their propensity to hide their pain behavior; and due to the complexity of the impact of a commercial production environment and interaction with handlers on physiological or endocrine measures. Electroencephalography (EEG), the measure of electrical activity in the brain, has been successfully demonstrated under general anaesthesia to objectively measure pain in livestock. However, the practicality of this application in the field is limited. We propose the use of EEG for objectively measuring pain in conscious lambs undergoing castration. Here we reveal that EEG can not only quantify the pain of castration, but also distinguish between anaesthetic interventions. This method has practical advantages that make it a useful measure of pain relief in sheep.

**Abstract:**

Australian sheep routinely undergo painful surgical husbandry procedures without anaesthesia or analgesia. Electroencephalography (EEG) has been shown to be a successful measure of pain in livestock under a general anaesthetic. The aim of this study was to compare this EEG model to that of conscious lambs undergoing castration with and without local anaesthesia. Sixteen merino crossbred ram lambs 6 to 8 weeks of age (13.81kg ± 1.97) were used in the study. Lambs were randomly allocated to 1 of 4 treatment groups: (1) Conscious EEG and surgical castration with no anaesthetic intervention (CON; *n* = 4); (2) Conscious EEG and surgical castration with pre-operative applied intra-testicular lignocaine injection (CON + LIG; *n* = 4); (3) surgical castration under minimal anaesthesia (MAM; *n* = 4); (4) and surgical castration with pre-operative lignocaine injection (2 mL lignocaine hydrochloride 20 mg/mL, under minimal anaesthesia (MAM + LIG; *n* = 4). Distinct differences in the EEG parameters Ptot, F50 and F95 between pre-and post-castration in conscious lambs were demonstrated in this study (*p* < 0.01). Further, CON and CON + LIG treatments were distinguishable using F50 and F95 measures (*p* = 0.02, *p* = 0.04, respectively). Significant changes in the EEG output of MAM animals were identified pre- to post-castration (*p* < 0.01). The EEG output of MAM and MAM + LIG were similar. EEG was successful in differentiating lambs treated with pain relief in a conscious state after castration by examining F50 and F95, which may suggest the suitability of conscious EEG pain measurement.

## 1. Introduction

Invasive husbandry procedures are routinely conducted on livestock across Australia as standard practice to improve production, health and wellbeing. Surgical husbandry procedures conducted on sheep include tail docking, castration, mulesing and ear tagging. Standard lamb marking procedures evoke varying pain mechanisms such as localised ischemic pain, or systemic inflammatory pain, which require different modes of measurement and treatment [1]. The Australian animal welfare standards and guidelines for sheep recommend the use of pain management when castrating sheep; however, it is only compulsory for lambs over 6 months of age [2] despite aversive behavioural and physiological responses having been observed in younger lambs from 5 days up to 45 days of age in response to castration [3,4,5,6,7]. Studies have shown electroencephalographic changes indicative of pain are observable in lambs from as young as 2 weeks of age [8,9]. Pain is notoriously difficult to quantify in animals due to the complexity of pain having both an emotional and physiological component. It is subject to individual experience, and in sheep, expression can be influenced by their stoic nature as a prey species. Further, measures of pain are often non-specific, and can be affected by non-painful stressors, such as handling or shearing [10]. The use of local anaesthetic has been shown to alleviate the pain of castration with studies demonstrating lignocaine, administered to the neck of the scrotum or directly into the testes, elicits significant decreases in both aversive behaviour and plasma cortisol concentrations in lambs during castration [11,12]. Despite this knowledge, pain measurement in livestock continues to be an area of industry concern with continued research focusing on improving methods to objectively measure pain. The pain associated with castration is well documented in lambs [12]. The constraints of large, extensively-run operations mean that suitable pain management for husbandry procedures that meet practical and economic requirements has not been established. Development of objective, robust pain measurement methods are essential for the implementation of successful pain management in sheep.

Electroencephalography (EEG) is the measurement of the summation of electrical current generated by the excitation of numerous pyramidal neurons in the cerebral cortex using electrodes [13]. Measuring cortical activity using EEG has been shown to correlate with reported pain in humans [14,15]. Previous work conducted by Chen et al. [15] found that EEG responses correlated to the reported pain experienced by participants. These studies have been used as a basis for continued development using EEG to measure pain. There are limited studies demonstrating the use of EEG to measure pain in conscious sheep, with varying results. Ong et al. [16] demonstrated a significant EEG response to electrical stimulation in sheep, whereby total power (Ptot) increased with increasing stimulus intensity. Another study investigating EEG in lambs undergoing several marking procedures, including castration, reported a decline in Ptot for more invasive procedures [10]. 

The minimal anaesthesia model (MAM) was developed as a method to assess the EEG of animals to measure pain responses without the subject perceiving pain, as well as reducing the confounding effects of other senses [17,18]. This model uses halothane gas-induced anaesthesia to measure the activation of nociceptive pathways without sensory distress to the animal [19]. This enables the collection of data during husbandry procedures without confounding signals from other senses, allowing pain signals to dominate.

Work conducted by Murrell et al [19] has demonstrated the use of the frequency parameters F50 (median frequency) and Ptot (total power) to evaluate the noxious stimulation of castration in horses. These parameters were further demonstrated to be indicative of pain in deer [20], cattle [21] and lambs [8] undergoing painful procedures. The MAM has shown consistent results for painful procedures, with increases in the F50 and a decrease in total power indicative of pain, which was attenuated by analgesia [8,20]. The model is limited; however, as subjects must remain anaesthetised for the duration of the procedure and for any data recording, which increases the risk to the subject, requires a high cost to maintain, and has practicality constraints for applications in the field setting where experimental procedures may require repeated measures across time. Objective measurements of post-procedural pain would provide valuable data on the efficacy of analgesic options. This, coupled with the cost associated with general anaesthesia, give merit to the use of EEG in conscious sheep. 

The objective of the current study was to examine the efficacy of electroencephalography as a measure of pain in lambs undergoing castration with or without lignocaine in both conscious and anaesthetised (MAM) states. 

## 2. Materials and Methods 

### 2.1. Animals

All experimental protocols were approved by the University of Sydney Animal Ethics Committee (AEC Approval No. 5832) 

Sixteen merino crossbred ram lambs 6 to 8 weeks of age (13.81kg ± 1.97) were used in the study. Lambs were sourced from the University of Sydney teaching flock, at Cobbitty, NSW. Prior to the experimental days, lambs were kept on pasture with dams and had previously undergone routine tail docking, ear tagging and vaccination. On the experimental days, lambs were transported by enclosed trailer 2.3km to the University Veterinary Teaching Hospital (Camden, NSW, Australia) where they were held in their group in an enclosed pen with straw bedding for the duration of the experiment. All lambs were examined prior to procedures by a veterinarian to ensure both testicles were present, and no signs of systemic disease were observed (scabby mouth, septic joints or omphalitis). Lambs were returned to dams at the conclusion of the study day following post-surgical assessment by the veterinarian. 

### 2.2. Treatments

The study was blocked over two days, with seven lambs treated on the first day and nine on the second day (Table 1). Lambs were randomly selected and allocated via computer-generated random numbers (Excel® version 16, © Microsoft 2018) to 1 of 4 treatment groups: (1) Conscious EEG and surgical castration with no anaesthetic intervention (CON; *n* = 4); (2) conscious EEG and surgical castration with pre-operative applied intra-testicular lignocaine injection (CON + LIG; *n* = 4); (3) surgical castration under minimal anaesthesia (MAM; *n* = 4); (4) and surgical castration with pre-operative lignocaine injection (2 mL lignocaine hydrochloride 20 mg/mL, Troy/Ilium) under minimal anaesthesia (MAM + LIG; *n* = 4). 

### 2.3. Castration Procedure

Castration was performed by two trained technicians using the same surgical technique. Lambs were moved from the holding pen to the surgery, where they were restrained in dorsal recumbency using a surgical positioning aide with limb restraint (Pawsitioner, Avail Concepts IIc, Sedona, AZ, USA) secured on a surgical table. A heat mat (EasyVet, NSW, Australia) was positioned between the restraint and surgical Table and set at 42 degrees Celsius. Animals in MAM + LIG and CON + LIG treatment groups were administered 1.5 mL lignocaine 20 mg/mL, into the spermatic cords and 0.5 mL into the scrotal skin using a 21G needle, 2 minutes prior to castration. Castration was performed using a sterile scalpel blade. The distal third of the scrotum was excised with one cut, and the testes exposed. Tension was placed on the spermatic cords using a twisting motion, and the testes were then removed using a scraping motion of the scalpel on the cords to reduce bleeding.

### 2.4. Anaesthesia Induction 

Anaesthesia was induced by a veterinary surgeon via an inhalation face mask using 4% halothane (Halothane BP, Pharmochem, Eagle Farm, QLD, Australia) vaporised in oxygen with oxygen flow at 20 mL/kg/min. Halothane Vapor setting was reduced between 1–3 % during the maintenance with oxygen flow at 10 mL/kg/min. Anaesthesia was maintained via facemask with a Surestream capnometer inserted prior (Capnostream™ Patient Monitor with Microstream™ Technology, MEDTRONIC© 2020© 2020, Minneapolis, MN, USA). Heart rate, end tidal CO_2_, temperature and respiration were monitored throughout the procedure by a veterinarian. Palpebral reflex and jaw tone were used to determine anaesthetic depth before EEG and ECG leads were connected.

### 2.5. Electroencephalography Recording 

Three 2 mm, monopolar needle electrodes (29 gauge) made from surgical steel (ADInstruments Ltd, Bella Vista, NSW, Australia) were used to record electroencephalograms for the lambs. Electrodes were attached in a 3-electrode montage adapted from Mayhew and Washbourne [22] as follows: The non-inverting electrode was placed in the midline over the frontal sinus, the inverting electrode over the right mastoid process, and the common electrode caudal to the occipital process. Electrode placement was kept consistent between treatment groups, with minor adjustments made for anatomical differences between lambs. EEG was recorded continuously at a sampling rate of 1 kHz, and analysed off-line after the completion of the experiment. An internal laptop camera was positioned to record movement artefacts, including blinking, chewing, respiration, vocalisations, and head turns. Video was recorded in real-time with the EEG output using the Video Capture add on (Lab Chart ADInstruments Ltd, Bella Vista, NSW, Australia). 

Electrocardiograms (ECG) were recorded using a three-electrode montage with an active electrode on the left fore-flank and a reference electrode on the inside of the right hind limb, with the ground electrode common to the EEG. Baseline EEG and ECG data were recorded for 10 minutes prior to castration and for 10 minutes post-castration. 

Following data collection, all animals were treated topically with 4.5 mL Tri-Solfen® topical anaesthetic (Bayer Animal Health, Pymble, NSW, Australia) by inserting the provided nozzle into the post-castration scrotal incision [23]. Lambs were also administered meloxicam subcutaneously at 1 mg/kg (Boehringer Ingelheim Pty Limited, North Ryde, NSW, Australia). Lambs were returned to the holding pen and monitored by a livestock veterinarian for the remainder of the experiment, up to a maximum of 5 hours including transport. Upon the completion of recording each day, the lambs were transported back to the home paddock and returned to the dams.

### 2.6. Data Analysis

EEG traces were manually inspected offline for movement artefacts in LabChart (LabChart Pro, version 8, AD Instruments Ltd, Bella Vista, NSW, Australia). This was further validated by comparison with the time-locked video footage of each procedure in LabChart. Data was then exported to MATLAB (version R2017b, MathWorks, Inc. Natick, Ma, USA), where 2-second epochs of data were fast Fourier transformed (FFT) and the frequency parameters F50, F95 and Ptot were extracted using purpose-specific code developed by the researchers. Results were then further analysed in Excel® (Microsoft Excel, version 16.13) and R (Version 1.1.447—© 2020© 2020–2018 RStudio, Inc. Boston, MA, USA). Restricted maximum likelihood test (REML) was used with fixed effects being treatment and time, and random effects of lamb and day. *p* values < 0.05 were considered statistically significant.

## 3. Results

The F50, F95, and Ptot outputs across treatment groups from pre- to post-castration are presented below (Table 2). There was a significant interaction between treatment and time with changes in EEG output observed pre- to post-castration for all parameters in all treatment groups, with the exception of F50 in MAM + LIG group. 

Significant increases in Ptot from pre- to post-castration were observed for CON, MAM and MAM + LIG (*p* < 0.05) with a decrease in CON + LIG (*p* = 0.005). There was a significant difference between F50 values from pre- to post-castration for CON, CON + LIG and MAM (*p* < 0.01). Significant differences pre-castration occurred between CON + LIG vs MAM; CON + LIG vs MAM + LIG; and MAM vs CON. Significant changes in F95 were observed between baseline recording pre-castration and post-castration for all treatment groups (*p* < 0.05). The F95 increased for MAM and MAM + LIG groups (*p* < 0.01 and *p* < 0.05, respectively) and decreased for CON group from pre- to post-procedure (*p* < 0.01). 

Results of differences between treatment groups pre- and post-castration are presented below (Table 3). Total power results showed no significant differences between any treatment group prior to castration. There were significant differences between anaesthetised and conscious animals for F50 and F95 pre-castration. There was no difference between MAM and MAM + LIG lambs for any parameter measured. CON and CON + LIG groups did not differ prior to castration; post-castration, these groups showed significant differences in F50 and F95.

## 4. Discussion

This study is the first to evaluate EEG as a measure of pain and analgesia comparing lambs undergoing castration in a conscious state to those under general anaesthesia. Changes in the EEG of conscious lambs from pre- to post-castration suggest a quantifiable pain response associated with the procedure. Additionally, we were able to discriminate between pain mitigation protocols in the EEG output of conscious lambs. Conscious lambs that were administered lignocaine prior to castration exhibited a consistent increase in F50 and a decrease in Ptot as compared to those that did not receive lignocaine. This study was unable to replicate previously-demonstrated trends in MAM groups of the established parameters F50, F95, and Ptot. The trend in conscious lambs shows potential for this method as an objective measure of pain and analgesic efficacy in sheep undergoing surgical castration. 

We have demonstrated the ability of EEG to identify and quantify pain in lambs. A change in EEG response post-castration from baseline was evident for all treatment groups, with the exception of MAM + LIG for F50. Previous studies have reported on changes in EEG output immediately following castration in lambs [8,9], tail docking in piglets [24], lamb marking [10], and pain responses of sheep to electrical stimulation [16]. Directional changes have been noted to differentiate painful and non-painful responses, with F50 reported as increasing after a painful event in animals under a general anaesthesia [8,9,19] and decreasing when conscious [10]. The F50 values for CON + LIG lambs were significantly greater than that of CON lambs’ post-castration. Prior to castration, there were no differences between the EEG responses of CON and CON + LIG lambs for F50, indicating minimal inter-animal variation. Therefore, the changes seen in the EEG post-castration can be attributed to a pain response to castration. Additionally, changes reflected in F50 could be indicative of anaesthetic efficacy. Castration is well documented to cause behavioural and endocrine changes indicative of pain [7], and the use of a local anaesthetic for pain relief during castration is widely accepted as a suitable practice to reduce associated pain and stress responses [25]. Lignocaine is the most commonly used local anaesthetic for husbandry procedures, it has been shown to successfully mitigate the pain response associated with procedures including castration [26,27,28]. In this study, the discrimination between anaesthetic interventions using F50 from EEG in conscious lambs may be indicative of anaesthetic efficacy.

We were not able to differentiate between MAM and MAM + LIG treatments post-castration using any parameter, limiting the usefulness of this method within the current study. Lambs castrated under minimal anaesthesia exhibited a significant increase in F50 and Ptot and a decrease in F95 post-castration; however these changes were not mitigated by lignocaine administration. Previous studies in lambs castrated using MAM reported an increase in the F50 of lambs post-castration [8,9] and in piglets undergoing castration [24]. 

This study demonstrated an increase in the Ptot values of CON lambs from pre- to post-castration. An increase in Ptot has been previously reported in conscious sheep undergoing a painful procedure [16], and in humans undergoing a cold pressure test, where EEG response correlated to the subjects descriptive pain [15]. This study showed Ptot changed significantly from the baseline in all treatment groups; however, directional changes were variable. Notably, CON lambs showed an increase post-castration, contrasting with the decrease shown in CON + LIG lambs. This suggests that Ptot may be a suitable parameter for the measurement of a painful procedure in conscious lambs; however, no significant differences were observed to differentiate between the two treatments using Ptot. This may limit the usefulness of this parameter in future studies as an indicator of analgesic treatment. The parameters F50 and F95 were able to both differentiate an effect of castration and use of lignocaine in conscious lambs, suggesting these may be useful measures moving forward. 

There are two approaches for the analysis of EEG data in sheep: (1) power separation into frequency bandwidths, as commonly used in human models [13]; and (2) the analysis of signal frequency trends by examining statistical descriptors, such as the mean and sum of frequencies. Power separation is an extrapolation from human EEG analysis involving the segmentation of signal output into pre-established bandwidth classifications, namely delta (0.5–3.5Hz), theta (4–8Hz), alpha (8–13Hz) and beta (13–30Hz). Analysis involves the examination of changes to the distribution of power between the bandwidths. This method has been extensively applied in human studies [13] and has had some limited application for animal studies [16,29,30]. However, care must be taken when making comparisons between human and animal models when using power separation analysis. With only limited application of this methodology in sheep, detailed interpretation based on human models can appear arbitrary [31]. 

The analysis of changes in signal frequency distribution trends has been used more extensively in studies on lambs [8,9]. In this method mathematical descriptors are used to analyse the EEG power spectrum; these commonly include the median frequency (F50), 95% spectral edge (F95) and the total power (Ptot), which give insight into the changing distribution of power in general (F50), and at higher (F95) and lower (Ptot) frequencies. Examining changes using the parameters F50, F95 and Ptot provides more generalised measures of EEG change relevant to nociception in sheep without unfounded assumptions for interpretation; therefore, this method was selected for data analysis in the current study. 

In the current study, it was found that F50 and F95 were the most comprehensive measure for conscious animals. This parameter was able to distinguish between lambs treated with lignocaine and those without (Table 3), as well as indicate a directional change in the median frequency after castration (Table 2). In future investigations of EEG of conscious lambs, F50 and F95 would be suitable measures to provide insight into the pain response. Though Ptot showed significant changes within a treatment group post-castration, this measure was unable to differentiate between treatments. Ptot and F95 were more reliable indicators than F50 of direction change post-castration within a treatment group for lambs in MAM and MAM + LIG groups. No differences were seen between these two treatment groups for any parameter. This may be due to the effects of the general anaesthesia. 

Halothane-induced anaesthesia is used in the MAM to minimize the suppressive effect on EEG response that general anaesthesia can result in when induced by other agents, including isoflurane and methoxyflurane [18]. Halothane has been shown to minimise EEG suppression in rats [32] and chickens [33] whilst still allowing responses to be observed. Despite this, dampening EEG could still be a factor impacting results between conscious and anaesthetised lambs in the present study due to the nature of the anaesthetic agency. It has also been suggested that a lack of uniformity of methodology across species, severity of procedures, and anaesthetic depth can influence the trends demonstrated in EEG [31]. 

Conscious EEG has practical experimental advantages over MAM that could benefit research into painful procedures conducted on sheep. These advantages include ease of measurement over an extended period of time and reduced risk to the animal. Based on the results reported in this study, conscious EEG could be implemented whilst still generating significant results. Inhalation anaesthesia is limited by the feasibility of repetitions in studies examining analgesic effects over time, and conscious EEG recording removes the need for lengthy or repeated anesthetization, allowing for repeated measures of pain response over time. The current study demonstrated significant changes in EEG occurred post-procedure and continued for at least 180 s. This is a relatively short period only addressing the immediate procedural pain of castration. Sustained changes in EEG from the baseline suggest the potential for longer-term efficacy studies of analgesic intervention in livestock for surgical husbandry procedures to be carried out. 

Movement artefact and environmental noise, such as that from electrical equipment, can impact on EEG output [29]. We observed an increase in outlying and spurious data points in the conscious EEG groups as compared to the MAM groups. However, this can be unavoidable, regardless of if MAM is used, due to movement caused as a response to procedural and handling manipulation, and as a result, small, 2–3 second sections of data may need to be discarded from analysis to avoid skewing results. Noise artefacts from movement during castration have meant procedural data cannot be collected; however, significant and meaningful results can still be obtained despite this, as observed in the present study, when comparing baseline values to those immediately post-procedure.

## 5. Conclusions

This study demonstrates promising results for the assessment of analgesic efficacy in lambs undergoing castration. This study was able to show measurable changes in the EEG output post-castration to distinguish between conscious lambs treated with local anaesthetic and those that were not. This method has the potential to be used to examine analgesic efficacy in lambs. Further research will be focused on using continuous EEG for repeated measurements over time. Further application of this method to other surgical husbandry procedures in sheep warrants investigation. 

## Figures and Tables

**Table 1:** The number of animals treated each day of the experiment for each treatment group.

Treatment	Day 1	Day 2
CON	*n* = 1	*n* = 3
CON + LIG	*n* = 1	*n* = 3
MAM	*n* = 3	*n* = 1
MAM + LIG	*n* = 2	*n* = 2

**Table 2:** Predicted means, with back-transformed values in parenthesis, for each treatment group before (Pre) and after (Post) castration. The table combines the measurement parameters of median frequency (F50), 95th percentile power (F95), and total power (Ptot).

Item	Time	CON (1)	CON + LIG (2)	MAM (3)	MAM + LIG (4)
F50	Pre	2.45 (11.61)	2.63 (13.81)	2.21 (9.08)	2.23 (9.33)
	post	2.41 (11.11)	2.68 (14.60)	2.28 (9.74)	2.24 (9.36)
	*p* value	<0.01	<0.01	<0.01	0.7493
F95	Pre	3.13 (22.87)	3.18 (23.96)	2.96 (19.29)	2.98 (19.73)
	post	3.08 (21.85)	3.17 (23.71)	3.00 (20.05)	3.00 (20.04)
	*p* value	<0.01	<0.01	<0.01	<0.05
Ptot	Pre	−9.44 (7.97 × 10^−5^)	−9.53 (7.25 × 10^−5^)	−8.97 (1.27 × 10^−4^)	−9.24 (9.71 × 10^−5^)
	post	−9.25(9.63 × 10^−5^)	−9.63 (6.59 × 10^−5^)	−8.88 (1.39 × 10^−4^)	−9.10 (1.12 × 10^−4^)
	*p* value	<0.01	<0.01	<0.01	<0.01

**Table 3:** *p*-values for pairwise comparisons of predicted means between treatment groups, within a time period, pre- or post-castration. Treatment groups are denoted as follows: Con (1), Con + Lig (2), MAM (3) and MAM + Lig (4). The table combines the measurement parameters of median frequency (F50), 95^th^ percentile power (F95) and total power (Ptot).

Item		Treatment Comparisons					
		1–2	1–3	1–4	2–3	2–4	3–4
F50	Pre	0.12	0.04	0.06	<0.01	<0.01	0.80
	Post	0.02	0.22	0.12	<0.01	<0.01	0.70
F95	Pre	0.22	<0.01	<0.01	<0.01	<0.01	0.55
	Post	0.04	0.03	0.03	<0.01	<0.01	0.99
Ptot	Pre	0.82	0.26	0.63	0.18	0.48	0.51
	Post	0.36	0.37	0.71	0.08	0.20	0.60

## References

[1] Coetzee J.F. (2011). A review of pain assessment techniques and pharmacological approaches to pain relief after bovine castration: Practical implications for cattle production within the United States. Appl. Anim. Behav. Sci..

[2] AHA (2014). Australian Animal Welfare Standards and Guidelines—Sheep. Tail Docking and Castration.

[3] Grant C. (2004). Behavioural responses of lambs to common painful husbandry procedures. Appl. Anim. Behav. Sci..

[4] Lester S.J., Mellor D.J., Holmes R.J., Ward R.N., Stafford K.J. (1996). Behavioural and cortisol responses of lambs to castration and tailing using different methods. N. Z. Vet. J..

[5] Molony V., Kent J.E., McKendrick I.J. (2002). Validation of a method for assessment of an acute pain in lambs. Appl. Anim. Behav. Sci..

[6] Molony V., Kent J.E., Robertson I.S. (1993). Behavioural responses of lambs of three ages in the first three hours after three methods of castration and tail docking. Res. Vet. Sci..

[7] Paull D., Lee C., Colditz I., Atkinson S., Fisher A. (2007). The effect of a topical anaesthetic formulation, systemic flunixin and carprofen, singly or in combination, on cortisol and behavioural responses of Merino lambs to mulesing. Aust. Vet. J..

[8] Johnson C.B., Stafford K.J., Sylvester S.P., Ward R.N., Mitchinson S., Mellor D.J. (2005). Effects of age on the electroencephalographic response to castration in lambs anaesthetised using halothane in oxygen. N. Z. Vet. J..

[9] Johnson C.B., Sylvester S.P., Stafford K.J., Mitchinson S.L., Ward R.N., Mellor D.J. (2009). Effects of age on the electroencephalographic response to castration in lambs anaesthetized with halothane in oxygen from birth to 6 weeks old. VAA.

[10] Jongman E.C., Morris J.P., Barnett J.L., Hemsworth P.H. (2000). EEG changes in 4-week-old lambs in response to castration, tail docking and mulesing. Aust Vet. J..

[11] Kent J.E., Molony V., Graham M.J. (1998). Comparison of methods for the reduction of acute painproduced by rubber ring castration or tail docking of week-old lambs. Vet. J..

[12] Mellor D.J., Molony V., Robertson I.S. (1991). Effects of castration on behaviour and plasma cortisol concentrations in young lambs, kids and calves. Res. Vet. Sci..

[13] Teplan M. (2002). Fundementals of EEG Measurement. Meas. Sci. Rev..

[14] Bromm B. (1984). Pain Measurement in Man: Neurophysiological Correlates of Pain.

[15] Chen A.C.N., Dworkin S.F., Haug J., Gehrig J. (1989). Topographic brain measures of human pain and pain responsivity. Pain.

[16] Ong R.M., Morris J.P., O’Dwyer J.K., Barnett J.L., Hemsworth P.H., Clarke I.J. (1997). Behavioural and EEG changes in sheep in response to painful acute electrical stimuli. Aust. Vet. J..

[17] Johnson C.B., Taylor P.M. (1997). Effects of alfentanil on the equine electroencephalogram during anaesthesia with halothane in oxygen. Res. Vet. Sci..

[18] Johnson C.B., Taylor P.M. (1998). Comparison of the effects of halothane, isoflurane and methoxyflurane on the electroencephalogram of the horse. Br. J. Anaesth..

[19] Murrell J.C., Johnson C.B., White K.L., Taylor P.M., Haberham Z.L., Waterman-Pearson A.E. (2003). Changes in the EEG during castration in horses and ponies anaesthetized with halothane. VAA.

[20] Johnson C.B., Wilson P.R., Woodbury M.R., Caulkett N.A. (2005). Comparison of analgesic techniques for antler removal in halothane-anaesthetized red deer (Cervus elaphus): Electroencephalographic responses. VAA.

[21] Gibson T.J., Johnson C.B., Stafford K.J., Mitchinson S.L., Mellor D.J. (2007). Validation of the acute electroencephalographic responses of calves to noxious stimulus with scoop dehorning. N. Z. Vet. J..

[22] Mayhew I.G., Washbourne J.R. (1990). A method of assessing auditory and brainstem function in horses. Br. Vet. J..

[23] Lomax S., Harris C., Windsor P.A., White P.J. (2017). Topical anaesthesia reduces sensitivity of castration wounds in neonatal piglets. PLoS ONE.

[24] Kells N.J., Beausoleil N.J., Sutherland M.A., Morrison R.M., Johnson C.B. (2017). Electroencephalographic assessment of oral meloxicam, topical anaesthetic cream and cautery iron for mitigating acute pain in pigs (Sus scrofa) undergoing tail docking. VAA.

[25] Mellor D.J., Stafford K.J. (2000). Acute castration and/or tailing distress and its alleviation in lambs. N. Z. Vet. J..

[26] Dinnis A.S., Stafford K.J., Mellor D.J., Bruce R.A., Ward R.N. (1997). Acute cortisol responses of lambs castrated and docked using rubber rings with or without a castration clamp. Aust. Vet. J..

[27] Stewart M., Beausoleil N.J., Johnson C.B., Webster J.R., Schütz K.E., Cox N., Stafford K.J. (2014). Do rubber rings coated with lignocaine reduce the pain associated with ring castration of lambs?. Appl. Anim. Behav. Sci..

[28] Sutherland M., Mellor D., Stafford K., Gregory N., Bruce R., Ward R., Todd S. (1999). Acute cortisol responses of lambs to ring castration and docking after the injection of lignocaine into the scrotal neck or testes at the time of ring application. Aust. Vet. J..

[29] Cwynar P., Kolacz R., Walerjan P. (2014). Electroencephalographic recordings of physiological activity of the sheep cerebral cortex. Pol. J. Vet. Sci..

[30] Haga H.A., Dolvik N.I. (2005). Electroencephalographic and cardiovascular variables as nociceptive indicators in isoflurane-anaesthetized horses. VAA.

[31] Murrell J.C., Johnson C.B. (2006). Neurophysiological techniques to assess pain in animals. J. Vet. Pharmacol. Ther..

[32] Orth M., Barter L., Dominguez C., Atherley R., Carstens E., Antognini J.F. (2005). Halothane and propofol differentially affect electroencephalographic responses to noxious stimulation. Br. J. Anaesth..

[33] McIlhone A.E., Beausoleil N.J., Kells N.J., Johnson C.B., Mellor D.J. (2018). Effects of halothane on the electroencephalogram of the chicken. Vet. Med. Sci..

