# Erratum: Harris, C.; White, P.J.; Mohler, V.L.; Lomax, S. Electroencephalography Can Distinguish between Pain and Anaesthetic Intervention in Conscious Lambs Undergoing Castration. *Animals* 2020, *10*, 428

**DOI:** 10.3390/ani10091498

**Published:** 2020-08-24

**Authors:** Charissa Harris, Peter John White, Virginia L. Mohler, Sabrina Lomax

**Affiliations:** 1 School of Life and Environmental Sciences, Faculty of Science, The University of Sydney, Sydney 2006, Australia; char2922@uni.sydney.edu.au; 2 Sydney School of Veterinary Science, Faculty of Science, The University of Sydney, Sydney 2006, Australia; jennie.mohler@sydney.edu.au (V.L.M.); p.white@sydney.edu.au (P.J.W.)

The authors wish to make the following corrections to this paper [1]:

## 1. Change in Simple Summary

In Line 10 of the Simple Summary, the phrase “This method has practical advantages that make it a useful measure of pain relief in sheep.” Has been edited to “This method has potential for objective measurement of pain and pain amelioration in sheep”. 

## 2. Change in the Abstract

In Line 1 of the Abstract, the sentence “Australian sheep routinely undergo painful surgical husbandry procedures without anaesthesia or analgesia” has been edited to “Australian sheep routinely undergo painful surgical husbandry procedures including surgical castration.” Moreover, additional sentences have been added at Line 2: “Commercially available pain mitigation drugs are becoming increasingly available for use on-farm, which has important implications for improved animal welfare. Pain in livestock can be difficult to quantify, due to the confounding effects of non-painful stressors on physiological and behavioural responses.”

## 3. Change to Funding

An additional line has been added: “AWI is grateful for its funding, which is primarily provided by Australian woolgrowers through a wool levy and by the Australian Government which provides a matching contribution for eligible R&D activities.”
